# Three Novel Marine Species of *Paracoccus*, *P. aerodenitrificans* sp. nov., *P. sediminicola* sp. nov. and *P. albus* sp. nov., and the Characterization of Their Capability to Perform Heterotrophic Nitrification and Aerobic Denitrification

**DOI:** 10.3390/microorganisms11061532

**Published:** 2023-06-09

**Authors:** Kun Zhang, Qi Zeng, Rouyun Jiang, Songbiao Shi, Jian Yang, Lijuan Long, Xinpeng Tian

**Affiliations:** 1 CAS Key Laboratory of Tropical Marine Bio-Resources and Ecology, Guangdong Key Laboratory of Marine Materia Medica, RNAM Center for Marine Microbiology, Sanya Institute of Oceanology, SCSIO, South China Sea Institute of Oceanology, Chinese Academy of Sciences, Guangzhou, 510301, China; 2 Sanya Institute of Ocean Eco-Environmental Engineering, Yazhou Scientific Bay, Sanya 572000, China; 3 University of Chinese Academy of Sciences, Beijing 100049, China

**Keywords:** *Paracoccus* novel species, polyphasic characterization, heterotrophic nitrification–aerobic denitrification

## Abstract

Heterotrophic nitrification–aerobic denitrification (HN-AD) is an efficient nitrogen removal process and the genus *Paracoccus* is one important group of the HN-AD bacteria. During an investigation of the microbial diversity in marine ranching of the Pearl River Estuary (PR China), three bacterial strains, designated SCSIO 75817^T^, SCSIO 76264^T^ and SCSIO 80058^T^, were isolated from sediments. Phylogenetic analyses based on 16S rRNA gene sequences indicated that the three strains belonged to the genus *Paracoccus* and their closest neighbors were *P. isoporae* DSM 22220^T^ (97.6–98.0%), *P*. *aurantiacus* CGMCC 1.13898^T^ (97.3–97.6%) and *P*. *xiamenensis* MCCC 1A16381^T^ (97.1–97.4%), respectively. The analysis results of 16S rRNA gene similarity, ANI, AAI and dDDH showed that the pairwise similarities between these three strains and their closest neighbors were 97.4–98.5%, 76.9–81.0%, 75.5–79.6% and 20.3–23.3%, respectively. Polyphasic taxonomic data of the phylogenetic, phenotypic and chemotaxonomic analyses indicate that these strains represent three novel species in the genus *Paracoccus*, for which the names *Paracoccus aerodenitrificans* sp. nov., *Paracoccus sediminicola* sp. nov. and *Paracoccus albus* sp. nov. are proposed, respectively. The study also demonstrated the heterotrophic nitrification–aerobic denitrification (HN-AD) ability of the novel species *P. aerodenitrificans* SCSIO 75817^T^. When it was aerobically cultivated at 28 °C using NH_4_^+^-N, NO_3_^−^-N and NO_2_^−^-N as the sole nitrogen sources, the nitrogen removal efficiencies were 73.4, 55.27 and 49.2%, respectively, and the maximum removal rates were 3.05, 1.82 and 1.63 mg/L/h, respectively. The results suggest that it has promising potential for wastewater treatment.

## 1. Introduction

The efficient removal of ammonia, nitrite and nitrate is critical for sewage treatment [1]. It has previously been widely accepted that aerobic-heterotrophic nitrogen removal occurs mainly via a heterotrophic nitrification-aerobic denitrification (HN-AD) pathway, in which ammonium is transformed into dinitrogen through a series of oxidation and reduction reactions, i.e., NH^4+^→ NH_2_OH → (NO_2_^−^ → NO_3_^−^ → NO_2_^−^) → NO → N_2_O → N_2_ [2]. Heterotrophic nitrification–aerobic denitrification (HN-AD) bacteria possess the remarkable capability of nitrification and denitrification simultaneously within one cell, which facilitates nitrogen removal from the environment [3]. HN-AD bacteria are not only employed in wastewater denitrification, but also in other areas, such as in waste gas treatment coupled with methane oxidation for denitrification. HN-AD bacteria were screened out from different environments, such as sludge, wastewater and wastewater treatment systems. These bacteria primarily belonged to genera such as *Paracoccus*, *Bacillus*, *Pseudomonas*, *Alcaligenes* and *Acinetobacter*, etc. [4]. Biological denitrification is considered to be the most efficient way to reduce nitrogen pollution with low operating costs [5]. Therefore, there is a necessity to screen more efficient HN-AD bacteria.

The genus *Paracoccus* was first proposed with the species type *Paracoccus denitrificans* by Davis et al. [6], and it widely exists in various environments, such as air [7], sewage [8], soil [9], horse blood [10], mangroves [11], reef-building coral [12], hydrothermal systems [13], seawater [14] and sediment [15]. These species also play important biological functions in their original environments, such as the fact that *P. zeaxanthinifaciens* and *P. haeundaensis* can produce astaxanthin, which has high antioxidant activity and great application values [16,17]. *P. denitrificans* can regulate sulfur metabolism [18] and nitrogen metabolism [19], and utilize molecular hydrogen in denitrification [20]. Previously, *P. versutus* KS293 has demonstrated its effective ability to remove nitrogen via denitrification processes [21]. Recently, a highly efficient HN-AD strain *P. denitrificans* HY-1 was isolated and investigated by Huan et al. [22]. Both strains KS293 and HY-1 revealed their good capability to carry out denitrification processes under both aerobic and anaerobic conditions.

During an investigation of the microbial diversity in marine ranching of the Pearl River Estuary, Guangdong Province (PR China), three *Paracoccus* novel species in the family *Rhodobacteraceae* were isolated from the sediment. We identified and proposed these three new species as being *Paracoccus aerodenitrificans* sp. nov., *Paracoccus sediminicola* sp. nov. and *Paracoccus albus* sp. nov., and also characterized the HN-AD capability of the strain SCSIO 75817^T^. This not only enhanced our knowledge of the diversity of the genus *Paracoccus*, but also provided valuable bacterial resources for the practical application of nitrogen pollution treatment.

## 2. Materials and Methods

### 2.1. Isolation, Maintenance and Screening

Three strains were isolated from sediment samples collected via Wanshan marine ranching in the Pearl River Estuary (22° N, 113.5° E, at depth of 18–20 m). The samples were serial diluted with sterile seawater and 200 μL suspension was transferred onto isolation media and incubated at 28 °C for 2 weeks. Strains SCSIO 76264^T^ and SCSIO 80058^T^ were picked from 20% marine agar (MA) medium and minimal salt (MS) medium, respectively. Strain SCSIO 75817^T^ was picked from 20% Reasoner’s 2A (R2A) medium. The pure cultures were preserved in glycerol suspensions (30%, *v*/*v*) at −80 °C. In order to determine the taxonomic status of three species, strain types *P. isoporae* DSM 22220^T^, *P. aurantiacus* CGMCC 1.13898^T^ and *P. xiamenensis* MCCC 1A16381^T^ were selected as the reference strains for polyphasic taxonomy tests under the same conditions. In addition, to screen the denitrification capability of these strains, the pure culture strains were inoculated onto the BTB plate at 28 °C and we subsequently conducted the nitrogen removal capability studies.

### 2.2. Morphology and Physiology

The morphological characteristics were observed using a transmission electron microscope (JEM-100CX-II; JEOL, Akishima, Tokyo, Japan) after incubation for 3 days at 28 °C on marine agar (MA) medium. Cell motility was examined using MA semisolid medium containing 0.4% agar. Gram-staining was performed according to the steps of the standard Gram reaction [23] combined with the KOH lysis test [24]. Anaerobic growth was determined after 2-week incubation at 28 °C using the GasPak EZ Anerobe Pouch system (BD). Cultural characteristics were observed after growth on various agar media for 2 weeks at 28 °C according to the methods described by Shirling and Gottlieb [25]. The hydrolysis of cellulose, starch, Tweens (20, 40, 60 and 80), gelatin liquefaction, coagulation and peptonization of milk, H_2_S production and nitrate reduction were observed using the methods described by Tindall et al. [26]. Catalase activity was tested by observing bubble production in hydrogen peroxide solution (*v*/*v*, 3%) and oxidase activity was tested using oxidase reagent (bioMérieux, Marcy-l’Étoile, France). The range of temperatures (4, 10, 15, 20, 28, 30, 35, 37, 40 and 45 °C) for growth was tested on MA medium. The growth pH range (pH 4.0−13.0 at intervals of 1.0 pH unit) was examined in marine broth (MB) at 28 °C using the buffer system described by Xu et al. [27]. Salt tolerance was measured on R2A agar supplemented with various concentrations of NaCl (0% and 1.0–10.0% at intervals of 1.0%). In addition, other biochemical characteristics and enzyme activities were further tested using the API ZYM (bioMérieux, Marcy-l’Étoile, France) and API 20NE (bioMérieux, Marcy-l’Étoile, France) kits according to the manufacturer’s instructions. Carbon and energy source utilization patterns were determined using Biolog GEN III (Biolog, CA, USA).

### 2.3. Chemotaxonomic Analysis

The cultures for chemotaxonomic analysis were obtained after incubation at 28 °C for 3 days on MA medium under an aerobic condition. The whole-cell fatty acids were separated, identified and quantified using the standard MIS Library generation software (Sherlock Version 6.1 MIDI, Clifton, NJ, USA; MIDI database: TSBA6, MIDI, Clifton, NJ, USA) according to the manufacturer’s instructions [28]. Quinones were extracted and examined following the procedures of Collins et al. [29] and Kroppenstedt [30]. The polar lipids were extracted using the methods described by Lechevalier [31], separated via two-dimensional TLC and identified as described by Minnikin et al. [32]. The first chromatographic direction was developed in chloroform/methanol/water (65: 25: 4, *v*/*v*), while the second direction was developed in a mixture of chloroform/methanol/acetic acid/water (80: 18: 12: 5, *v*/*v*).

### 2.4. 16S rRNA Gene Phylogeny

Genomic DNA extraction and PCR amplification of the 16S rRNA gene were carried out following the methods described by Li et al. [33] with the universal primers 27F (5′-AGAGTTTGATCCTGGCTCAG-3′) and 1492R (5′- GGTTACCTTGTTACGACTT-3′) [34]. The 16S rRNA gene sequences were analyzed in EzBioCloud using BLAST (https://www.ezbiocloud.net/, accessed on 7 December 2016) [35]. Multiple alignments with reference sequences were carried out using CLUSTAL X 1.83 [36]. Phylogenetic trees were reconstructed based on three algorithms: neighbor-joining [37], maximum-likelihood [38] and maximum-parsimony [39], using MEGA version 11 [40]. The method used to compute evolutionary distances was Kimura’s two-parameter [41]. The topologies of the phylogenetic trees were evaluated via bootstrap analyses after 1000 replications [42].

### 2.5. Genome Analysis

Genomic DNA were extracted using HiPure Bacterial DNA kits (Magen, Guangzhou, China) and the complete genomes were sequenced using the PacBio RS II platform of Tianjin Biochip Company (Tianjin, China). Denovo genome assembly was performed using the HGAP4 (Pacific Biosciences, SMRT Link V6.0; San Francisco, CA, USA) tool, following the Hierarchical Genome Assembly Process (HGAP) [43]. The prediction of rRNA and tRNA information was made using Barrnap and tRNA-scan [44], respectively. Functional annotation of the genomes was performed via rapid annotation using Subsystems Technology (RAST) and visualized using the SEED viewer [45]. The metabolic pathways in a single bacterium were reconstructed using the online tool KEGG Mapper [46]. Secondary metabolism was scanned using antiSMASH (version 6.1.1) [47]. The DNA G+C content was determined from a whole genome sequence. The average nucleotide identity (ANI) was calculated using the ANI Calculator (https://www.ezbiocloud.net/tools/ani/, accessed on 7 December 2016) [48]. The digital DNA-DNA hybridization (dDDH) value was calculated using the Genome-to-Genome Distance Calculator 3.0 (https://ggdc.dsmz.de/ggdc.php, accessed on 11 October 2021) [49]. The average amino acid identity (AAI) was estimated using the AAI calculator tool (http://enve-omics.ce.gatech.edu/, accessed on 27 March 2016) [50]. Genome phylogenetic trees were constructed using RAxML [51] based on the 120 single-copy genes using the GTDB-Tk software toolkit [52], and fast bootstrapping [53] was used to generate the support values in the trees. The whole genome and orthologous gene analysis were compared using OrthoVenn 2 [54]. Synteny analysis of the genomes with their closest related strain types were conducted using the progressive Mauve tool [55].

### 2.6. Nitrogen Removal Performance of the Isolated Strain

Different nitrogen sources were used to assess the nitrogen removal capacity of strains, and (NH_4_)_2_SO_4_, NaNO_2_ or NaNO_3_ were, respectively, added into NM or DM with an initial nitrogen concentration of 105 mg/L. The NM consisted of 2.85 g/L sodium citrate, 0.64 g/L NaNO_3_, 0.8 g/L KH_2_PO_4_, 0.4 g/L MgSO_4_·7H_2_O, 0.4 g/L NaCl, 0.04 g/L FeSO_4_·7H_2_O and 0.03 g/L CaCl_2_, at pH 7–7.2. Moreover, 0.64 g/L NaNO_3_ and 0.52 g/L NaNO_2_ were added into DM1 and DM2 media, respectively, instead of (NH_4_)_2_SO_4_ as the sole nitrogen source in the NM [56]. Meanwhile, to evaluate the simultaneous nitrification and denitrification capacity of the strains, equal amounts of NaNO_3_ and (NH_4_)_2_SO_4_ (calculated by N, 105 mg/L) were added to SNDM medium. Briefly, 2% (*v/v*) inoculation of cell suspension was added to the medium, and the experiment was conducted at 28 °C and 160 rpm for 72 h. Culture solutions (5 mL) were collected every 12 h, and the corresponding value of OD600, as well as the values of nitrogen, including ammonium (NH_4_^+^-N), nitrate (NO_3_^−^-N), nitrite (NO_2_^−^-N) and total nitrogen (TN), were determined, respectively [57]. 

### 2.7. Analytical Methods of Nitrogen Removal

After centrifugation of the culture samples at 8000 rpm for 10 min, NH_4_^+^-N, NO_2_^−^-N, NO_3_^−^-N and TN were measured using Nessler’s reagent spectrophotometry method, the N-(1-naphthalene)-diaminoethane photometry method, phenol disulfonic acid photometry method and alkaline potassium persulfate method, respectively [58]. The optical density of the bacterial solution was measured using a microplate reader at a wavelength of 600 nm. The nitrogen removal efficiency (RE) was calculated using the following formula: RE (%) = (C_0_ − C_1_)/ C_0_ × 100%, and the formula of nitrogen removal rate (RR) was RR (mg/L/h) = (C_1_ − C_2_)/ t. C_1_ and C_2_ were the initial and the final concentrations (after t h cultivation) of TN, NH_4_^+^-N, NO_3_^−^-N and NO_2_^−^-N, respectively. All experiments included three replicates, and the results were presented as means ± SD.

## 3. Results and Discussion

### 3.1. Phenotypic Characteristics

Three strains’ colonies were tiny, smooth, convex and cream-white after 3 days of incubation on MA medium. The cells of them were aerobic, Gram-negative, non-motile, non-flagellated and rod-shaped (SCSIO 75817^T^, 0.8–1.5 × 2.0–2.4 µm; SCSIO 76264^T^, 0.7–0.8 × 1.1–2.0 µm; SCSIO 80058^T^, 0.6–0.7 × 1.6–1.7 µm; Figure S1). The strain SCSIO 75817^T^ grew well on MA, NA, LB, TSA and R2A, but did not grow on ISP 2 agar. The strains SCSIO 76264^T^ and SCSIO 80058^T^ grew well on MA and R2A, but did not grow on ISP 2, NA, TSA and LB agar. SCSIO 75817^T^ had a higher range of growth adaptability at 4–40 °C (optimum, 28 °C), pH 6–10 (optimum, 7) and in the presence of 0–9% (*w*/*v*) NaCl (optimum, 0–1%). The growth of SCSIO 76264^T^ was observed at 4–40 °C (optimum, 28 °C), pH 6–8 (optimum, 7) and 0–6% NaCl (optimum, 0–1%). The growth of SCSIO 80058^T^ was observed at 8–37 °C (optimum, 28 °C), pH 6–9 (optimum, 7) and in the presence of 0–6% (*w*/*v*) NaCl (optimum, 0–1%). All of the strains were negative for the hydrolysis of starch, cellulose, casein, Tween 80 and the production of indole, and were positive for aesculin, oxidase and catalase. Strains SCSIO 76264^T^ and SCSIO 80058^T^ were positive for the hydrolysis of Tweens 20 and 40, but strain SCSIO 75817^T^ was only positive for Tween 40. SCSIO 75817^T^ and SCSIO 80058^T^ were positive for the hydrolysis of gelatin, but SCSIO 76264^T^ was negative. The detailed results of the physiological and biochemical results of three novel strains and the closest neighbors are presented in Table 1, Table S1 and the species description.

### 3.2. Chemotaxonomic Characterization

The major cellular fatty acids (>10%) of strains SCSIO 75817^T^ (86.8%), SCSIO 76264^T^ (84.0%) and SCSIO 80058^T^ (78.2%) were identified as C_18:1_ *ω*7*c*, which was consistent with the reference strains *P. isoporae* DSM 22220^T^ (85.4%), *P*. *aurantiacus* CGMCC 1.13898^T^ (77.8%) and *P*. *xiamenensis* MCCC 1A16381^T^ (84.2%). The detailed cellular fatty acids of these new strains and related strain types are shown in Table S2. The predominant respiratory ubiquinone was Q-10, which was consistent with the typical characteristics of the genus *Paracoccus*. Three strains contained DPG, PE, PG and PC as their diagnosable polar lipids, only different at PL, L or GL (Figure S2). However, the PE was absent in their reference strains. 

### 3.3. Phylogenetic Analysis

**Figure 1:**
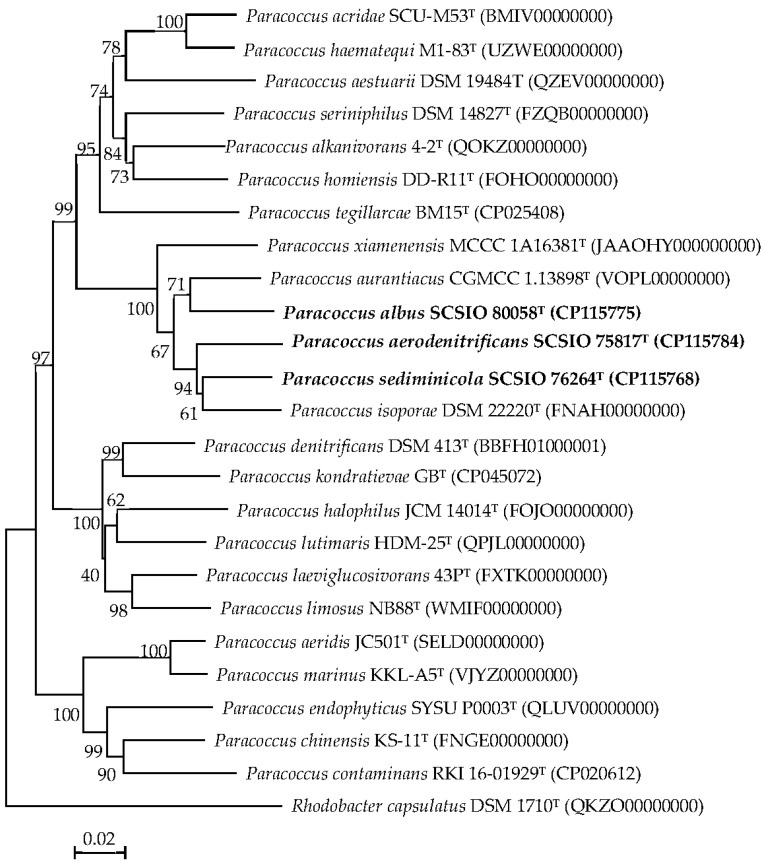
Phylogenetic analysis based on genome sequences of strains SCSIO 75817^T^, SCSIO 76264^T^ and SCSIO 80058^T^ with their closest related taxa. *Rhodobacter capsulatus* DSM 1710^T^ was added as an outgroup. Bar: 0.02 substitutions per nucleotide position.

Three nearly full-length 16S rRNA gene sequences of the three strains were obtained. Pairwise sequence analyses of the 16S rRNA genes indicated that the closest strains were *P. isoporae* DSM 22220^T^ (similarities: 97.6–98.0%), *P*. *aurantiacus* CGMCC 1.13898^T^ (similarities: 97.3–97.6%) and *P*. *xiamenensis* MCCC 1A16381^T^ (similarities: 97.1–97.4%). The neighbor-joining phylogenetic tree (Figure S3) also indicated that these three strains clustered together and should be members of the genus *Paracoccus*. Remarkably, these similarity values were well below the threshold (98.65%) for prokaryotes species demarcation, as suggested by Kim et al. [59]. Neighbor-joining phylogenetic trees supported by the three tree-making algorithms based on the 16S rRNA gene sequences indicated that all three of the strains clustered entirely with their closest neighbors and clearly formed three distinct lineages in the genus *Paracoccus*. In addition, phylogenetic trees based on maximum-likelihood (Figure S4) and maximum-parsimony algorithms (Figure S5) also verified the close relationships. The genome phylogenetic tree (Figure 1) based on 120 single-copy genes also showed that these three strains clearly formed three distinct lineages as in the reconstructed 16S rRNA gene trees. Therefore, these three strains should be assigned to the genus *Paracoccus* and did not belong to any known species.

### 3.4. Whole Genome Sequence Analysis and Annotation 

The genome sizes of SCSIO 75817^T^, SCSIO 76264^T^ and SCSIO 80058^T^ were 3,318,305 bp, 3,175,232 bp and 3,318,811 bp, with DNA G+C content of 60.6%, 64.1% and 60.1%, respectively. All of these strains included one circular chromosome and 4–6 circular plasmids. They contained 6 rRNA (2 16S, 2 23S and 2 5S) and 47–50 tRNA genes, with a total gene count of 3256, 3040 and 3299, respectively. All of the 16S rRNA genes in each strain were consistent with genomic-PacBio and Sanger sequencing. The detailed gene compositions listed in Table S3 clearly reveal that three new taxa differ in a few features compared to their close neighbors of *P. isoporae* DSM 22220^T^, *P*. *aurantiacus* CGMCC 1.13898^T^ and *P*. *xiamenensis* MCCC 1A16381^T^.

The genomes of SCSIO 75817^T^, SCSIO 76264^T^ and SCSIO 80058^T^ contained 3150, 2973 and 3183 genes of the clusters of orthologous groups (COG), respectively. The analysis of COG function revealed that the genomes of the three candidate strains have richer COG categories of “Energy production and transformation” and “Replication, recombination and repair” than the reference strains, while “Chromatin structure and dynamics” was present only in the genome of SCSIO 76264^T^ (Table S4). According to the SEED viewer, the putative genes annotated in strains SCSIO 75817^T^ (2447 genes), SCSIO 76264^T^ (1395 genes) and SCSIO 80058^T^ (1506 genes) via the RAST pipeline were distributed into 437, 300 and 301 subsystems, respectively (Figure S6). These subsystem features were mainly classified as carbohydrates, amino acids and derivatives, fatty acids, lipids and isoprenoids, cofactors, vitamins, prosthetic groups and pigments. All three strains contained stress response coding genes within five subcategories as osmotic stress, oxidative stress, detoxification, periplasmic stress and unknown subcategories. The number of stress response coding genes of strain SCSIO 75817^T^ (111) was far higher than that of strains SCSIO 76264^T^ (41) and SCSIO 80058^T^ (53). Additionally, strain SCSIO 75817^T^ also contained the genes’ encoding stress responses within two subcategories of cold shock (2 genes) and heat shock (16 genes) that were absent in others. Therefore, combining this with the temperature range test, it was more adaptable to temperature than the other two strains. 

These three new strains showed the obvious differences in the secondary metabolite synthesis gene clusters. Strain SCSIO 75817^T^ had biosynthetic gene clusters (BGCs) encoding for homoserine lactone, non-ribosomal peptide synthetases (NRPS) and ectoine. Strain SCSIO 76264^T^ contained a beta-lactone-containing protease inhibitor, a homoserine lactone and an ectoine synthesizing cluster. The BGCs of strain SCSIO 80058^T^ included a NRPS-like (non-ribosomal peptide synthetase) fragment and an ectoine-synthesizing cluster. Both of the reference strains *P*. *aurantiacus* CGMCC 1.13898^T^ and *P*. *xiamenensis* MCCC 1A16381^T^ contained one non-ribosomal peptide metallophores cluster, while one unspecified ribosomally synthesized and post-translationally modified peptide product (RiPP-like) gene cluster existed only in strain *P*. *xiamenensis* MCCC 1A16381^T^. Each of these three strains contained two complete KEGG metabolic pathway modules, which were different from each other. The pathway module of beta-oxidation, the acyl-CoA synthesis (M00086) of fatty acid metabolism, was identified in all three strains’ genomes. The assimilatory nitrate reduction pathway (M00531) was only discovered in the genome of SCSIO 75817^T^. The beta-oxidation (M00087) of the fatty acid metabolism of lipid metabolism was discovered in the genome of strains SCSIO 76264^T^ and SCSIO 80058^T^, but was absent in strain SCSIO 75817^T^ (Table S5).

### 3.5. Comparative Genomic Analysis

The average nucleotide identity (ANI), average amino acid identity (AAI) and digital DNA-DNA hybridization (dDDH) results showed that the pairwise similarities between the strains SCSIO 75817^T^, SCSIO 76264^T^ and SCSIO 80058^T^ and their neighbors of *P*. *isoporae* DSM 22220^T^, *P*. *aurantiacus* CGMCC 1.13898^T^ and *P*. *xiamenensis* MCCC 1A16381^T^ were 76.9–81.0% (ANI < 95–96%), 75.5–79.6% (AAI < 95%) and 20.3–23.3% (dDDH < 70%), respectively. The values of ANI, AAI and dDDH between the three strains SCSIO 75817^T^, SCSIO 76264^T^ and SCSIO 80058^T^ were 77.6–79.4%, 76.3–78.0% and 20.8–21.1%, respectively. All of these values were below the recommend thresholds for new species (ANI, 95–96%; dDDH, 70%; AAI, 95–96%;) [60]. All of the values of ANI, dDDH and AAI between these three strains and other closely related *Paracoccus* species are shown in Table 2. 

**Figure 2:**
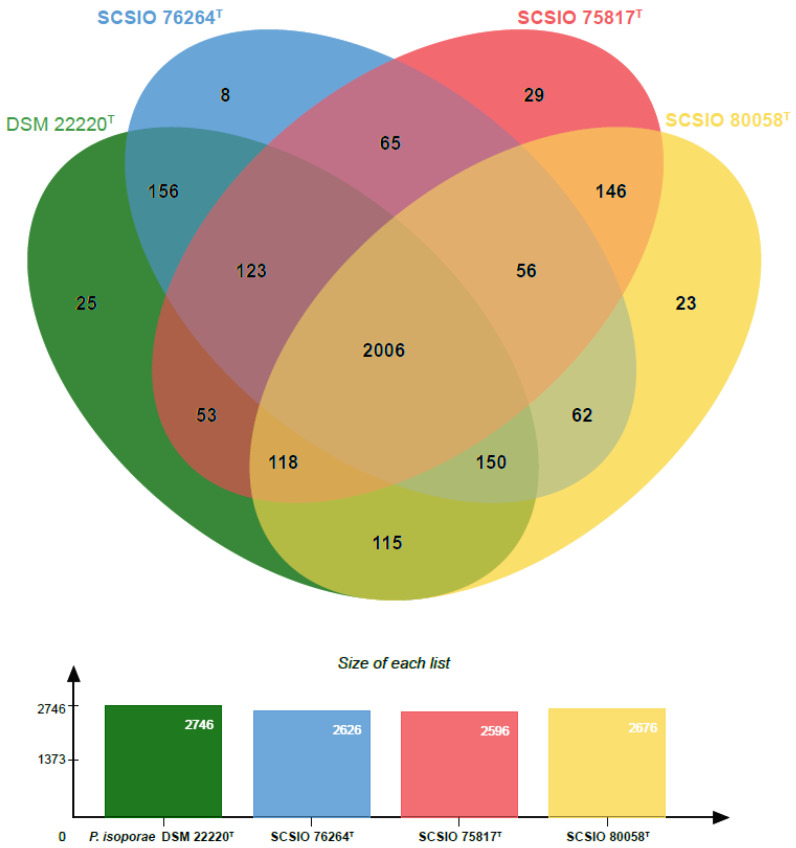
Venn diagram representing the core orthologs and unique genes for strains SCSIO 75817^T^, SCSIO 76264^T^ and SCSIO 80058^T^ and the most closely related strain type *P. isoporae* DSM 22220^T^.

**Figure 3:**
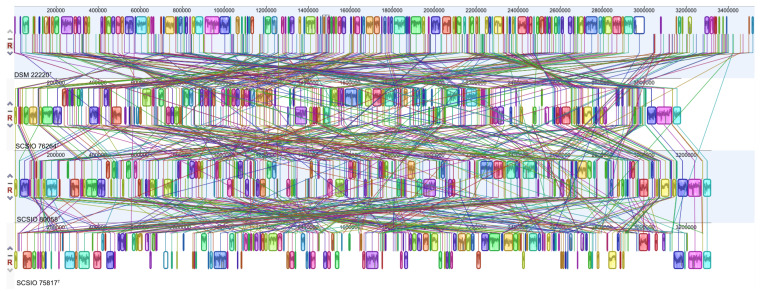
Genome alignment between strains SCSIO 75817^T^, SCSIO 76264^T^ and SCSIO 80058^T^ and the most closely related strain type *P. isoporae* DSM 22220^T^. Each contiguously colored locally collinear block (LCB) represents a region without rearrangement of the homologous backbone sequence.

OrthoVenn2 analysis (Figure 2) for a specific orthologous relationship among strains SCSIO 75817^T^ (2596 clusters), SCSIO 76264^T^ (2626 clusters) and SCSIO 80058^T^ (2676 clusters) and the most closely related strain type *P. isoporae* DSM 22220^T^ (2746 clusters) indicated that they formed a total of 3135 clusters; among them, 1156 were orthologous clusters (at least present in two strains) and 1979 were single-copy gene clusters. The four strains shared 2006 clusters, with the three largest classes being associated with molecular function (49), hydrolase activity (39), transporter activity (37), oxidoreductase activity (36), ion binding (31), nucleic acid binding (30) and transferase activity (29). A comparison of the orthologous gene numbers revealed that *P. isoporae* DSM 22220^T^ shared a higher number of gene clusters with SCSIO 76264^T^ (2609) than with SCSIO 75817^T^ (2506) and SCSIO 80058^T^ (2568), which reflected that strain DSM 22220^T^ has more of a close relation to strain SCSIO 76264^T^ than the other two strains. For a comprehensive genome comparison, synteny block analyses were performed on well-conserved large segment sequences between three new strains using the progressive Mauve tool. The results revealed that three strains shared many locally collinear blocks with their reference genomes, but all of them exhibited large-scale genome rearrangements (Figure 3). 

**Figure 4:**
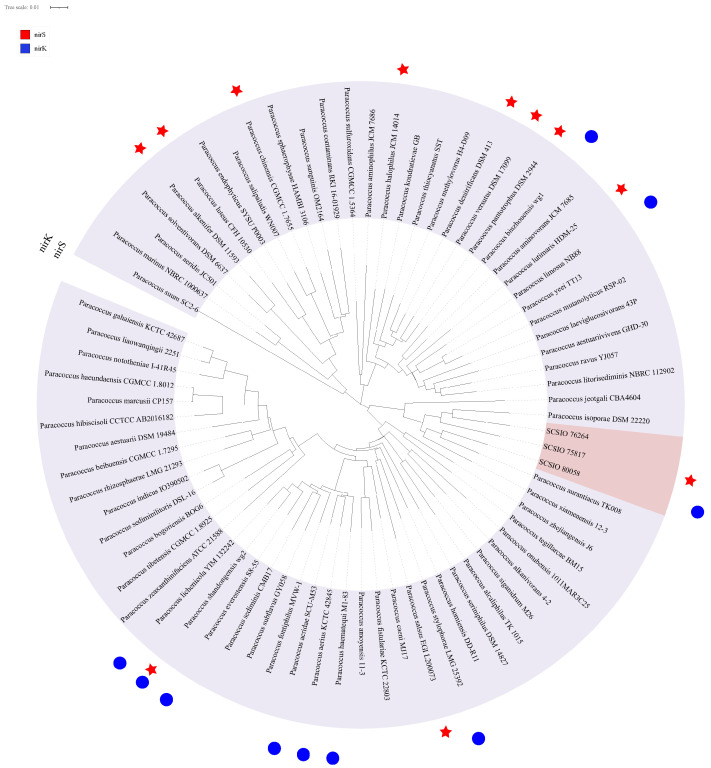
The function genes *nir*S and *nir*K of denitrification in 74 genomes of genus *Paracoccus*. Star: *nir*S gene; circle: *nir*K gene. Tree scale: 0.01.

Denitrification plays a crucial role in the nitrogen cycle within the marine environment. The strains SCSIO 75817^T^ and SCSIO 80058^T^ possessed 10 and 14 genes encoding denitrification, respectively. However, these denitrification genes were not found in SCSIO 76264^T^ (Table S6). These genes, such as *nir*S, *cnor*B, *cnor*C, *nor*D, *nor*E, *nor*Q, *nos*R, *nnr*S, etc, encode many nitrite reductases, nitrous oxide reductases and accessory proteins. In addition, strain SCSIO 75817^T^ was positive for nitrate reduction via the API 20NE kit’s preliminary test. The genome annotation results also revealed that strain SCSIO 75817^T^ had many nitrate and nitrite reduction reductase coding genes, and the assimilatory nitrate reduction complete pathway of nitrogen metabolism, which reflected the ability to assimilate nitrate into ammonia. The nitrite reductase genes (*nir*S and *nir*K) are usually considered to be key functional enzyme genes participating in the process of HN–AD. *nir*S is the gene of coding cytochrome cd1 nitrite reductase and *nir*K is the gene of coding copper-containing nitrite reductase. The key functional genes *nir*S and *nir*K were analyzed in validly published species of *Paracoccus*. By analyzing 74 genomes, 20 species were found to possess nitrite reductase genes, and the species *P. shandongensis* had both the *nir*S and *nir*K genes (Figure 4). *P. versutus* and *P. denitrificans* have previously been shown to have the denitrification function that can effectively remove nitrogen [18,21]. The gene *nir*S of strain SCSIO 75817^T^ encoded 596 amino acids’ nitrite reductase with the highest sequence similarity to *P. denitrificans* at 76.78%. Additionally, the strain SCSIO 75817^T^ also encoded nitrate reductase (MopB) of Nitrate-R-NapA-like with 100% similarity. These results also reflect the presence of many denitrifying bacteria in *Paracoccus* and the fact that they may play an important role in nitrogen removal in the environment.

### 3.6. Heterotrophic Nitrification–Aerobic Denitrification (HN-AD) Ability of Strain 75817^T^

**Figure 5:**
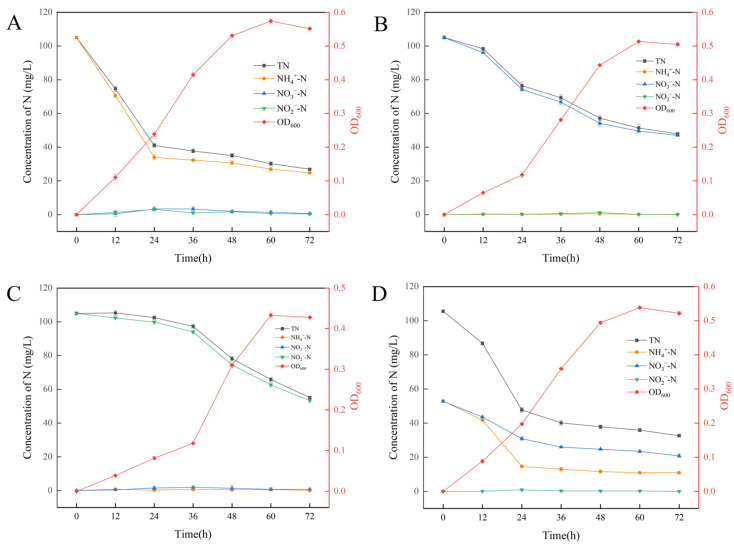
Bacterial growth and nutrient removal in (**A**) ammonium removal medium (NM), (**B**) nitrate removal medium (DM1), (**C**) nitrite removal medium (DM2) and (**D**) mixed ammonia nitrate removal medium (SNDM). Values are means ± SD for three replicates.

The three new species were tested for the denitrification function on a BTB agar plate, and only SCSIO 75817^T^ showed the obvious positive results (Figure S7A). Strain SCSIO 75817^T^ could be cultured in liquid medium with different sole nitrogen sources (Figure S7B). Heterotrophic nitrification performance was examined using (NH_4_)_2_SO_4_ as the sole nitrogen source. The variations in cell density and nitrogen concentrations during NH_4_^+^-N removal are shown in Figure 5A. 76.40% of NH_4_^+^-N was reduced after 72 h and the NH_4_^+^-N concentration decreased dramatically from 105 mg/L to 33.97 mg/L with the maximum removal rate being 3.05 mg/L/h between 12 and 24 h. The OD_600_ value reached the maximum level of 0.574 at 60 h and then decreased to 0.551 at 72 h. No obvious accumulation of NO_2_^−^-N and NO_3_^−^-N was observed during the NH_4_^+^-N removal process. The removal efficiency of TN in the whole denitrification process was 74.36%. These results demonstrate the outstanding nitrification efficiency and low level of intermediate products under aerobic conditions.

The aerobic denitrification ability was examined using different nitrogen sources; DM as the basal medium was used with NaNO_3_ and NaNO_2_ as the sole nitrogen source, respectively (Figure 5B,C). The NO_3_^−^-N concentration decreased from 105 mg/L to 46.97 mg/L at 72 h and the nitrogen removal efficiency was 55.27%. The maximum removal rate was 1.82 mg/L/h between 12 and 24 h. During the NO_3_^−^-N removal process, NO_2_^−^-N was accumulated and then consumed, and the maximum accumulation was 1.2 mg/L. The OD_600_ value increased to a maximum level of 0.513 at 60 h and then declined slightly at 72 h. When NO_2_^−^-N was used as the sole N source, NO_2_^−^-N decreased slowly from 0 to 36 h and decreased dramatically from 36 to 48 h with 93.92 mg/L to 74.39 mg/L. It is generally inferred that the cytotoxicity of nitrate made the strain SCSIO 75817^T^ take a longer time to adapt to the high-concentration N environments. 49.2% of NO_2_^−^-N was reduced at 72 h with the maximum removal rate of 1.63 mg/L/h between 36 and 48 h. The OD_600_ value reached the maximum level of 0.43 at 60 h, and subsequently declined slightly at 72 h. In addition, the OD_600_ value decreased dramatically from 36 to 48 h, which was consistent with the time of drastic change. The pattern of TN removal was consistent with that of NO_3_^−^-N and NO_2_^−^-N. The removal efficiency of TN in the whole denitrification process was 54.43% and 47.52%, respectively, when nitrate and nitrite were used as the nitrogen sources.

The simultaneous nitrification and aerobic denitrification ability of strain SCSIO 75817^T^ were examined using a mixture of N sources ((NH_4_)_2_SO_4_ and NaNO_3_) (Figure 5D). With the growth of strain SCSIO 75817^T^, a decrease in NH_4_^+^-N occurred immediately and the maximum removal rate was 3.25 mg/L/h between 12 and 24 h. The removal efficiency of NH_4_^+^-N and NO_3_^−^-N in the whole HN-AD process was 79.23% and 60.6%, respectively. Unlike the results observed in the sole N source experiments, NO_3_^−^-N showed the higher removal efficiency with the mixed N sources. This was consistent with the results that adding NH_4_^+^-N could promote the NO_3_^−^-N removal efficiency of *Arthrobacter arilaitensis* Y-10 [61]. The maximum removal rate of NO_3_^−^-N was 1.06 mg/L/h. According to our observations, NH_4_^+^-N was recognized as the primary N source for the strain SCSIO 75817^T^, which was removed at a faster rate than NO_3_^−^-N. In all of the results, the TN value was slightly higher than the sum of NH_4_^+^-N, NO_3_^−^-N and NO_2_^−^-N, and this may be the reason that some other forms of soluble nitrogen are produced during cell metabolism. The above results show that the strain SCSIO 75817^T^ is highly capable of HN-AD and can utilize all three N sources, with NH_4_^+^-N being the most suitable source of nitrogen for its growth.

### 3.7. Ecology

Members of the genus *Paracoccus* are versatile and highly adaptable in the marine environment, such as reef-building coral, hydrothermal systems, seawater and sediment. These bacteria have the ability to grow under various conditions, including aerobic, anaerobic, chemoorganotrophic or chemolithoautotrophic environments, and play an important role in nature, especially in nutrient and energy cycles. Nitrate assimilation is the main process of the nitrogen cycle, in which nitrate is first incorporated into the cells via an active transport system and then reduced to nitrite and ammonium by the enzyme nitrate and nitrite reductases [62]. The nitrogen reduction function has been found frequently in the genus *Paracoccus,* such as the *P. thiophilus* strain LSL 251 [63] and the *P. denitrificans* DYTN-1 [64]. In this research, three novel species of the genus *Paracoccus* were isolated from marine ranching environments. Genome analysis of the subsystem features of nitrogen metabolism revealed that strains SCSIO 75817^T^ and SCSIO 80058^T^ have the coding genes for denitrification involving nitrite reductase, nitrous oxide reductase and accessory coding genes, such as *nir*S, *cnor*B, *cnor*C, *nor*D, *nor*E, *nor*Q, *nos*R, *nnr*S, etc. Additionally, the KEGG genome annotation results revealed that strain SCSIO 75817^T^ had the assimilatory nitrate reduction complete pathway of nitrogen metabolism, which reflected the ability to assimilate nitrate into ammonia. We also found that strain SCSIO 75817^T^ was a novel HN-AD species. All three strains contained stress response coding genes within five subcategories: osmotic stress, oxidative stress, detoxification, periplasmic stress and unknown subcategories. In particular, strain SCSIO 75817^T^ also contained two subcategories of stress response coding genes: cold shock and heat shock. Cold shock genes coded CspA and CspB proteins and heat shock genes coded DnaK, DnaJ, GrpE protein, etc. The Csp family protein allowed the bacteria to acquire different specificities in response to temperature changes. The protein DnaK forms chaperone machinery with co-chaperones DnaJ and GrpE, which participates actively in the response to hyperosmotic and heat shock by preventing the aggregation of stress-denatured proteins and by disaggregating proteins. Combining this with the temperature range test, the strain SCSIO 75817^T^ not only had HN-AD ability, but could also adapt to different environmental changes. Therefore, according to these analysis results, the new species, SCSIO 75817^T^, exhibits a distinct nitrogen removal capacity to keep the marine ranching environment healthy. 

### 3.8. Description of Three Novel Species

*Paracoccus aerodenitrificans* sp. nov. (ae.ro. de.ni.tri’fi.cans. Gr. n. aer air; M.L. inf. denitrificare, denitrifying; M. L. part. adj. *aerodenitrificans*, the ability of aerobic denitrification).

The cells are aerobic, Gram-negative, non-motile, non-flagellated and rod-shaped (0.8 − 1.5 × 2.0 − 2.4 µm). They grow well on MA, NA, LB, TSA and R2A, but not on ISP 2 agar. Their growth occurs in 4–40 °C, pH 6–10 and in the presence of 0−9% (*w*/*v*) NaCl. The catalase and oxidase tests are positive. They are positive for the hydrolysis of Tweens 40 and 60 and gelatin, but negative for the hydrolysis of casein, starch, cellulose, coagulation and peptonization of milk, Tweens 20 and 80 and H_2_S. The predominant respiratory ubiquinone was Q-10. The major cellular fatty acid is C_18:1_ *ω*7*c* (86.8%). The polar lipid profile contains DPG, PE, PG, PC, one PL and two GLs. The strain type is SCSIO 75817^T^ (=MCCC 1K07995^T^ = KCTC 92497^T^).

*Paracoccus sediminicola* sp. nov. (se.di.mi.ni′co.la. L. n. sedimen -inis sediment; L. suff. -cola (from L. n. incola) inhabitant, dweller; N.L. n. *sediminicola*, sediment dweller).

The cells are aerobic, Gram-negative, non-motile and rod-shaped with a size of approximately 0.7–0.8 μm in width and 1.1–2.0 μm in length without flagellum. They grow well on MA and R2A, but not on ISP 2. Their growth occurs in 4–40 °C, pH 6–8 and 0–6% NaCl. They are positive for catalase and oxidase and the hydrolysis of Tweens 20 and 40, but negative for the hydrolysis of casein, starch, gelatin, cellulose, coagulation and peptonization of milk, Tweens 60 and 80 and H_2_S. The predominant respiratory ubiquinone was Q-10. The major cellular fatty acid is C_18:1_ *ω*7c (84.0%). The polar lipid profile contains DPG, PE, PG, PC, two GLs and two Ls. The strain type is SCSIO 76264^T^ (=MCCC 1K07996^T^ = KCTC 92501^T^).

*Paracoccus albus* sp. nov. (al’bus. L. masc. adj. *albus*, white).

The cells are Gram-negative and non-motile, flagella were not observed and they are rod-shaped with sizes of 1.6–1.7 µm in length and 0.6–0.7 µm in width. They grow well on MA and R2A, but not on ISP 2, NA, TSA or LB agar. Their growth occurs in 8–37 °C, pH 6–9 and in the presence of 0−6% (*w*/*v*) NaCl. They are positive for catalase and oxidase, the hydrolysis of Tweens 20 and 40 and gelatin, but negative for the hydrolysis of casein, starch, cellulose, coagulation and peptonization of milk, Tweens 60 and 80 and H_2_S. The predominant respiratory ubiquinone was Q-10. The major cellular fatty acid is C_18:1_ *ω*7c (78.2%). The polar lipid profile contains DPG, PE, PG, PC and two GL. The strain type is SCSIO 80058^T^ (=MCCC 1K07970^T^ = KCTC 92496^T^). 

## 4. Conclusions

These results of the 16S rRNA gene similarity, ANI, AAI, dDDH phylogenetic, phenotypic and chemotaxonomic analyses indicate that strains SCSIO 75817^T^, SCSIO 76264^T^ and SCSIO 80058^T^ represent three novel species in the genus *Paracoccus* of the family *Rhodobacteraceae*, for which the names *Paracoccus aerodenitrificans* sp. nov., *Paracoccus sediminicola* sp. nov. and *Paracoccus albus* sp. nov. are proposed. We also found that the novel species *P. aerodenitrificans* SCSIO 75817^T^ had obvious HN-AD capability, the nitrogen removal efficiencies were 73.4, 55.27 and 49.2%, respectively, and the maximum removal rates were 3.05, 1.82 and 1.63 mg/L/h, respectively, with NH_4_^+^-N, NO_3_^−^-N and NO_2_^−^-N as the sole nitrogen sources when it was aerobically cultivated at 28 °C. Thus, the strain SCSIO 75817^T^ may have good application prospects for wastewater purification.

## Figures and Tables

**Table 1:** Phenotypic characteristics that differentiate strains SCSIO 75817^T^, SCSIO 76264^T^ and SCSIO 80058^T^ from their phylogenetically related species.

Characteristic	1	2	3	4	5	6
Isolation source	Sediment	Sediment	Sediment	Coral	Hydrothermal	Seawater
Colony color	Creamy-white	Creamy-white	Creamy-white	Creamy-white	Orange	Creamy-white
Temperature (°C)	4–40	4–40	8–37	8–40	10–40	4–37
pH	6–10	6–8	6–9	7–10	6–8	6–10
NaCl (%, *w*/*v*)	0–9	0–6	0–6	3–7	0–5	0–6
Nitrate reduction	+	−	−	−	−	+
D-Glucose fermentation	−	−	−	+	−	−
PNPG	−	+	+	+	+	+
Hydrolysis of						
Tweens 20	−	+	+	−	+	−
Tweens 40	+	+	+	+	−	−
Tweens 60	+	−	−	−	−	−
Gelatin	+	−	+	−	−	−
Assimilation of						
D-Glucose	+	−	+	+	+	+
L-Arabinose	−	+	−	−	+	−
D-mannose	+	−	+	−	+	+
D-Mannitol	−	+	−	−	+	+
D-Maltose	+	+	+	+	+	−
Potassium gluconate	−	−	−	+	−	+
Enzymatic activities:						
Esterase (C4)	−	+	+	+	+	+
Esterase lipase (C8)	−	+	+	+	−	−
Trypsin	−	−	−	−	−	+
*α*-Chymotrypsin	−	−	+	+	−	+
*β*-Galactosidase	−	−	−	+	−	−
*α*-Glucosidase	+	−	−	+	−	−
*β*-Glucosidase	−	+	+	+	+	+
*N*-Acetyl-*β*-glucosaminidase	−	−	+	−	+	+
*α*-Mannosidase	−	−	−	+	−	−

Note: SCSIO 75817^T^; 2, SCSIO 76264^T^; 3, SCSIO 80058^T^; 4, *P. isoporae* DSM 22220^T^; 5, *P*. *aurantiacus* CGMCC 1.13898^T^; 6, *P*. *xiamenensis* MCCC 1A16381^T^. All data were obtained from this study. All strains were negative for the hydrolysis of starch, cellulose, casein, Tween 80, production of indole, H_2_S and coagulation and peptonization of milk, and positive for aesculin, oxidase and catalase. In API 20NE tests, all strains were positive for arginine dihydrolase, urease and aesculin hydrolysis. In the API ZYM kits, all strains were positive for alkaline phosphatase, leucine arylamidase, valine arylamidase, cystine arylamidase, acid phosphatase, naphthol-AS-BI- phosphohydrolase and *α*-glucosidase.

**Table 2:** Results of ANIb, AAI and dDDH between genomes of strains SCSIO 75817^T^, SCSIO 76264^T^ and SCSIO 80058^T^ and their most closely related species.

Strains	ANI (%)			dDDH (%)				AAI (%)	
	1	2	3	1	2	3	1	2	3
1	*	79.4	77.7	*	22.1	21.1	*	78.0	76.3
2	79.4	*	77.6	22.1	*	20.8	78.0	*	76.8
3	77.7	77.6	*	21.1	20.8	*	76.3	76.8	*
4	78.7	81.0	77.5	21.6	23.3	20.8	77.2	79.6	76.8
5	76.9	77.9	78.2	20.3	21.0	21.5	75.5	75.9	78.0
6	77.2	78.4	78.9	20.3	20.9	21.0	75.5	75.8	77.2

Note: SCSIO 75817^T^; 2, SCSIO 76264^T^; 3, SCSIO 80058^T^; 4, *P. isoporae* DSM 22220^T^; 5, *P*. *aurantiacus* CGMCC 1.13898^T^; 6, *P*. *xiamenensis* MCCC 1A16381^T^. *, 100% similarity.

## Data Availability

The GenBank/EMBL/DDBJ accession numbers for the 16S rRNA gene sequences of strains SCSIO 75817^T^, SCSIO 76264^T^ and SCSIO 80058^T^ are OQ259307, OQ259505 and OQ259507, and their genome sequences are CP115784, CP115768 and CP115775, respectively.

## Supplementary Materials

The following supporting information can be downloaded at https://www.mdpi.com/article/10.3390/microorganisms11061532/s1: Table S1: Energy source utilization of SCSIO 75817^T^, SCSIO 76264^T^ and SCSIO 80058^T^. Table S2: Fatty acid comparison of strains SCSIO 75817^T^, SCSIO76264^T^ and SCSIO 80058^T^ and the closely related neighbors. Table S3: General genome features of the three new species and the reference strains. Strains: 1, SCSIO 75817^T^; 2, SCSIO 76264^T^; 3, SCSIO 80058^T^; 4, *P. isoporae* DSM 22220^T^; 5, *P*. *aurantiacus* CGMCC 1.13898^T^; 6, *P*. *xiamenensis* MCCC 1A16381^T^. Table S4: Comparison of COG functions of three new species and the reference strains. Strains: 1, SCSIO 75817^T^; 2, SCSIO 76264^T^; 3, SCSIO 80058^T^; 4, *P. isoporae* DSM 22220^T^; 5, *P*. *aurantiacus* CGMCC 1.13898^T^; 6, *P*. *xiamenensis* MCCC 1A16381^T^. Table S5: The differences in the KEGG metabolic pathways of the genomes of strains SCSIO 75817^T^, SCSIO 76264^T^ and SCSIO 80058^T^. Table S6: Denitrification-related genes of strains SCSIO 75817^T^, SCSIO 76264^T^ and SCSIO 80058^T^ in nitrogen metabolism. Figure S1: Transmission electron microscopy images show the cell morphology of strains SCSIO 75817^T^ (a), SCSIO 76264^T^ (b) and SCSIO 80058^T^ (c) after being grown on MA medium at 28 °C for 3 days. a: Bar: 1 μm; b: bar: 0.5 μm; c: bar: 0.5 μm. Figure S2: Two-dimensional thin-layer chromatography of polar lipids of strains SCSIO 75817^T^ (a), SCSIO 76264^T^ (b) and SCSIO 80058^T^ (c). The first direction developed in chloroform/methanol/water (65:25:4, *v*/*v*/*v*), and the second developed in chloroform/methanol/acetic acid/water (80:12:15:4, *v*/*v*/*v*/*v*). DPG, diphosphatidylglycerol; PE, phosphatidylethanolamine; PG, phosphatidylglycerol; PC, phospatidyl choline; PL, unidentified phospholipid; GL, unidentified glycolipid; L, unidentified lipid. Figure S3: Neighbor-joining phylogenetic tree based on nearly-complete 16S rRNA gene sequences of strains SCSIO 75817^T^, SCSIO 76264^T^ and SCSIO 80058^T^ with their closest related taxa. *R. capsulatus* DSM 1710^T^ was used as an outgroup. Numbers at nodes indicate the percentage of 1000 bootstrap replicates. Only bootstrap values above 50% are shown. Bar: 0.005 substitutions per nucleotide position. Figure S4: The maximum-likelihood phylogenetic tree based on 16S rRNA gene sequences of strains SCSIO 75817^T^, SCSIO 76264^T^ and SCSIO 80058^T^ with related taxa. Numbers at nodes indicate the bootstrap values (>50%) based on 1000 replications. *Rhodobacter capsulatus* DSM 1710^T^ (jgi.1048965) was an outgroup. Bar: 0.005 substitutions per nucleotide position. Figure S5: The maximum-parsimony phylogenetic tree shows relationships between strains SCSIO 75817^T^, SCSIO 76264^T^, SCSIO 80058^T^ and related taxa based on 16S rRNA gene sequences. Numbers at nodes refer to bootstrap values (1000 replicates; only values > 50% are shown). *Rhodobacter capsulatus* DSM 1710^T^ was used as an outgroup. Figure S6: The subsystem category number of genes in strains SCSIO 75817^T^, SCSIO 76264^T^ and SCSIO 80058^T^ detected via RAST annotation server. Figure S7: Screening of denitrifying bacteria using BTB plate (A) and culture of different sole nitrogen sources (B) including NH_4_^+^-N, NO_3_^−^-N and NO_2_^−^-N.

## Abbreviations

MA: marine agar; R2A, Reasoner’s 2A agar; NA, nutrient agar; LB, Luria–Bertani agar; TSA, tryptic soy agar; ISP 2, International Streptomyces Project 2 medium; ANI, average nucleotide identity; dDDH, digital DNA–DNA hybridization; DPG, diphosphatidylglycerol; PG, phosphatidylglycerol; PC, phosphatidylcholine; PE, phosphatidylethanolamine; GL, unidentified glycolipid; PL, unknown phospholipid; L, unknown lipid.
