# Supplementary material for: Three Novel Marine Species of Paracoccus, P. aerodenitrificans sp. nov., P. sediminicola sp. nov. and P. albus sp. nov., and the Characterization of Their Capability to Perform Heterotrophic Nitrification and Aerobic Denitrification
Source: Microorganisms. 2023 Jun 9;11(6):1532. doi: 10.3390/microorganisms11061532 (PMC10302349; doi:10.3390/microorganisms11061532)
Supplement: Supplementary file 1 [file microorganisms-11-01532-s001.zip › microorganisms-2429529-supplementary.pdf]

# Supplementary Material

# Three Novel Marine Species of *Paracoccus*, *P. aerodenitrificans* sp. nov., *P. sediminicola* sp. nov. and *P. albus* sp. nov., and the Characterization of Their Capability to Perform Heterotrophic Nitrification and Aerobic Denitrification

**Kun Zhang [1,2,3,†], Qi Zeng [1,2,3,†], Rouyun Jiang [1,2,3], Songbiao Shi [1,2,3], Jian Yang [1,2,*], Lijuan Long [1,2] and Xinpeng Tian [1,2,*]**

[1] CAS Key Laboratory of Tropical Marine Bio-Resources and Ecology, Guangdong Key Laboratory of Marine Materia Medica, RNAM Center for Marine Microbiology, Sanya Institute of Oceanology, SCSIO, South China Sea Institute of Oceanology, Chinese Academy of Sciences, Guangzhou, 510301, China
[2] Sanya Institute of Ocean Eco-Environmental Engineering, Yazhou Scientific Bay, Sanya 572000, China
[3] University of Chinese Academy of Sciences, Beijing 100049, China
* Correspondence: yangjian@scsio.ac.cn (J.Y.); xinpengtian@scsio.ac.cn (X.T.); Tel.: +86-20-89023378 (J.Y. & X.T.)
† These authors contributed equally to this work.

**Table S1.** Energy sources utilization of SCSIO 75817$^T$, SCSIO 76264$^T$ and SCSIO 80058$^T$.

| SCSIO 75817$^T$ | SCSIO 76264$^T$ | SCSIO 80058$^T$ |
|---|---|---|
| It can utilize dextrin, D-maltose, D-trehalose, D-cellobiose, D-turanose, D-raffinose, *β*-methyl-D-glucoside, D-salicin, *N*-acetyl-D-glucosamine, *α*-D-glucose, D-mannose D-fructose, inosine, D-sorbitol, D-mannitol, D-arabitol, myo-inositol, D-fructose-6-$PO_4$, D-aspartic acid, gelatin, glycyl-L-prolin, L-alanine, L-arginine, L-aspartic acid, L-glutamic acid, pectin, D-gluconic acid, *p*-hydroxy-phenylacetic acid, methyl pyruvate, D-lactic acid methyl ester, L-lactic scid, citric acid, Tween 40, *γ*-amino-butryric acid *α*-hydroxy-butyric acid, *β*-hydroxy-D,L-butyric acid, *α*-keto-butyric acid. | It can utilize dextrin, D-maltose, D-trehalose, D-cellobiose, sucrose, D-turanose, *α*-D-lactose, D-salicin, *N*-acetyl-D-glucosamine, *N*-acetyl-*β*-D-mannosamine, *N*-acetyl-D-galactosamine, *α*-D-glucose, D-fructose, D-galactose, D-fucose, L-fucose, inosine, D-mannitol, D-arabitol, myo-inositol, glycerol, D-aspartic acid, L-alanine, L-aspartic acid, L-glutamic acid, L-pyroglutamic acid, pectin, D-gluconic acid, mucic acid, *p*-hydroxy-phenylacetic acid, methyl pyruvate, D-lactic acid methyl ester, L-lactic scid, citric acid, *α*-keto-glutaric acid, D-malic acid, L-malic acid, Tween 40, *γ*-amino-butryric acid *α*-hydroxy-butyric acid, *β*-hydroxy-D,L-butyric acid, *α*-keto-butyric acid, acetoacetic acid, propionic acid, acetic acid. | It can utilize dextrin, D-maltose, D-trehalose, D-cellobiose, gentiobiose, sucrose, D-turanose, D-melibiose, *β*-methyl-D-glucoside, D-salicin, *N*-acetyl-*β*-D-mannosamine, *N*-acetyl-D-galactosamine, *α*-D-glucose, D-mannose, D-fructose, D-Galactose, D-fucose, L-fucose, L-rhamnose, D-sorbitol, D-mannitol, D-arabitol, myo-inositol, glycerol, D-glucose-6-$PO_4$, D-fructose-6-$PO_4$, D-aspartic acid, gelatin, glycyl-L-proline, L-alanine, L-arginine, L-aspartic acid, L-histidine, L-pyroglutamic acid, pectin, D-galacturonic acid, L-galactonic acid lactone, D-gluconic acid, D-glucuronic acid, mucic acid, quinic acid, D-lactic acid methyl ester, L-lactic scid, citric acid, *α*-keto-glutaric acid, D-malic acid, L-malic acid, Tween 40, *γ*-amino-butryric acid *α*-hydroxy-butyric acid, *β*-hydroxy-D,L-butyric acid, acetoacetic acid, propionic acid, acetic acid. |

**Table S2.** Fatty acid comparison of strains SCSIO 75817$^T$, SCSIO76264$^T$, SCSIO 80058$^T$ and the closely related neighbors.

Strains: 1, SCSIO 75817$^T$; 2, SCSIO 76264$^T$; 3, SCSIO 80058$^T$; 4, DSM 22220$^T$; 5, MCCC 1A16381$^T$; 6, CGMCC 1.13898$^T$. All data were obtained from this study. Values are percentages of total fatty acids. The major fatty acids (greater than 10%) are shown in bold. TR, less than 1%. – , data not available.

| Fatty acid | 1 | 2 | 3 | 4 | 5 | 6 |
|---|---|---|---|---|---|---|
| $C_{16:0}$ | TR | TR | TR | TR | TR | 2.0 |
| $C_{18:0}$ | 5.2 | 5.5 | 8.1 | 6.3 | 6.0 | **12.0** |
| $C_{19:0}$ | TR | TR | TR | TR | 2.4 | TR |
| $C_{10:0}$ 3OH | 2.9 | 2.1 | 2.8 | 2.7 | 2.7 | 2.9 |
| Anteiso-$C_{15:0}$ | – | 1.8 | TR | TR | TR | TR |
| Anteiso- $C_{17:0}$ | – | 2.5 | TR | TR | TR | TR |
| $C_{18:1}$ $\omega$7c | **86.8** | **84.0** | **78.2** | **85.4** | **84.2** | **77.8** |
| $C_{18:1}$ $\omega$7c 11-methyl | – | TR | 1.2 | TR | – | 1.4 |
| $C_{19:0}$ cyclo $\omega$8c | – | – | 6.9 | TR | 1.1 | 1.5 |
| $C_{20:1}$ $\omega$7c | 2.2 | 1.1 | 1.1 | 1.7 | TR | TR |

**Table S3.** General genome features of the three new species and the reference strains. Strains: 1, SCSIO 75817$^T$; 2, SCSIO 76264$^T$; 3, SCSIO 80058$^T$; 4, *P. isoporae* DSM 22220$^T$; 5, *P. aurantiacus* CGMCC 1.13898$^T$; 6, *P. xiamenensis* MCCC 1A16381$^T$.

| **Parameter** | **1** | **2** | **3** | **4** | **5** | **6** |
|---|---|---|---|---|---|---|
| GenBank accession number | CP115780 | CP15768 | CP11575 | FNAH00000000 | VOPL00000000 | JAAOHY00000000 |
| Genome size (bp) | 3,318,305 | 3,175,232 | 3,318,811 | 3,523,473 | 3,788,400 | 3,643,702 |
| Number of Contigs | 1 | 1 | 1 | 31 | 42 | 21 |
| No. of genes | 3,256 | 3,040 | 3,299 | 3,427 | 3,786 | 3,576 |
| Plasmid | 4 | 6 | 4 | 0 | 0 | 0 |
| 16S rRNA | 2 | 2 | 2 | 1 | 1 | 1 |
| 23S rRNA | 2 | 2 | 2 | 1 | 2 | 1 |
| 5S rRNA | 2 | 2 | 2 | 1 | 2 | 1 |
| tRNA | 50 | 47 | 47 | 44 | 45 | 46 |
| G+C content (mol%) | 60.6 | 64.1 | 60.1 | 65.8 | 62.5 | 63.9 |

**Table S4.** Comparison of COG functions of three new species and the reference strains. Strains: 1, SCSIO 75817$^T$; 2, SCSIO 76264$^T$; 3, SCSIO 80058$^T$; 4, *P. isoporae* DSM 22220$^T$; 5, *P. aurantiacus* CGMCC 1.13898$^T$; 6, *P. xiamenensis* MCCC 1A16381$^T$.

| Code | 1 | 2 | 3 | 4 | 5 | 6 | Description |
|---|---|---|---|---|---|---|---|
| B | 0 | 1 | 0 | 0 | 0 | 0 | Chromatin structure and dynamics |
| C | 212 | 210 | 209 | 188 | 194 | 199 | Energy production and conversion |
| D | 44 | 44 | 33 | 26 | 26 | 46 | Cell cycle control, cell division, chromosome partitioning |
| E | 315 | 347 | 377 | 289 | 283 | 322 | Amino acid transport and metabolism |
| F | 75 | 79 | 79 | 75 | 80 | 91 | Nucleotide transport and metabolism |
| G | 191 | 214 | 253 | 201 | 232 | 277 | Carbohydrate transport and metabolism |
| H | 104 | 97 | 110 | 78 | 80 | 124 | Coenzyme transport and metabolism |
| I | 130 | 161 | 166 | 101 | 115 | 190 | Lipid transport and metabolism |
| J | 178 | 184 | 187 | 164 | 171 | 212 | Translation, ribosomal structure and biogenesis |
| K | 228 | 209 | 263 | 169 | 212 | 228 | Transcription |
| L | 296 | 201 | 259 | 148 | 159 | 119 | Replication, recombination and repair |
| M | 206 | 195 | 182 | 188 | 175 | 199 | Cell wall/membrane/envelope biogenesis |
| N | 44 | 48 | 43 | 32 | 32 | 36 | Cell motility |
| O | 154 | 125 | 140 | 126 | 132 | 159 | Posttranslational modification, protein turnover, chaperones |
| P | 226 | 192 | 250 | 203 | 188 | 191 | Inorganic ion transport and metabolism |
| Q | 93 | 117 | 125 | 84 | 69 | 83 | Secondary metabolites biosynthesis, transport and catabolism |
| R | 0 | 0 | 0 | 0 | 0 | 180 | General function prediction only |
| S | 642 | 641 | 690 | 959 | 1195 | 248 | Function unknown |
| T | 89 | 82 | 89 | 80 | 83 | 104 | Signal transduction mechanisms |
| U | 92 | 96 | 67 | 52 | 71 | 38 | Intracellular trafficking, secretion, and vesicular transport |
| V | 44 | 31 | 40 | 49 | 51 | 86 | Defense mechanisms |
| W | 0 | 0 | 0 | 0 | 0 | 1 | Extracellular structures |
| X | 0 | 0 | 0 | 0 | 0 | 38 | Mobilome: prophages, transposons |
| Z | 2 | 1 | 0 | 0 | 0 | 1 | Cytoskeleton |

**Table S5.** The differences in the KEGG metabolic pathways of the genomes of strains SCSIO 75817$^T$, SCSIO 76264$^T$ and SCSIO 80058$^T$.

| Complete KEGG metabolic pathways | 1 | 2 | 3 |
|---|---|---|---|
| **Energy metabolism** | | | |
| **Nitrogen metabolism** | | | |
| M00531 Assimilatory nitrate reduction, nitrate => ammonia | + | - | - |
| **Lipid metabolism** | | | |
| **Fatty acid metabolism** | | | |
| M00086 beta-Oxidation, acyl-CoA synthesis | + | + | + |
| M00087 beta-Oxidation | - | + | + |

**Table S6.** Denitrification related genes of strains SCSIO 75817$^T$, SCSIO 76264$^T$ and SCSIO 80058$^T$ in nitrogen metabolism.

| Genes | Gene function | SCSIO 75817$^T$ | SCSIO 76264$^T$ | SCSIO 80058$^T$ |
|---|---|---|---|---|
| ***nir*S** | **Cytochrome cd1 nitrite reductase (EC:1.7.2.1)** | + | - | - |
| ***nir*K** | **Copper-containing nitrite reductase (EC 1.7.2.1)** | - | - | + |
| ***nir*V** | **Nitrite reductase accessory protein NirV** | - | - | + |
| ***cnor*B** | **Nitric-oxide reductase subunit B (EC 1.7.99.7)** | + | - | + |
| ***cnor*C** | **Nitric-oxide reductase subunit C (EC 1.7.99.7)** | + | - | + |
| ***nor*E** | **Nitric oxide reductase activation protein NorE** | + | - | + |
| ***nor*D** | **Nitric oxide reductase activation protein NorD** | + | - | + |
| ***nor*Q** | **Nitric oxide reductase activation protein NorQ** | + | - | + |
| ***nos*Z** | **Nitrous-oxide reductase (EC 1.7.99.6)** | - | - | + |
| ***nos*D** | **Nitrous oxide reductase maturation protein NosD** | - | - | + |
| ***nos*F** | **Nitrous oxide reductase maturation protein NosF (ATPase)** | - | - | + |
| ***nos*Y** | **Nitrous oxide reductase maturation transmembrane protein NosY** | - | - | + |
| ***nos*L** | **Nitrous oxide reductase maturation protein, outer-membrane lipoprotein NosL** | - | - | + |

| | | | | |
|---|---|---|---|---|
| *nos*X | Nitrous oxide reductase maturation periplasmic protein NosX | + | - | - |
| *nos*R | Nitrous oxide reductase maturation protein NosR | + | - | + |
| *nnr*S | NnrS protein involved in response to NO | + | - | + |
| *nnr*U | NnrU family protein, required for expression of nitric oxide and nitrite reductases (Nir and Nor) | + | - | - |

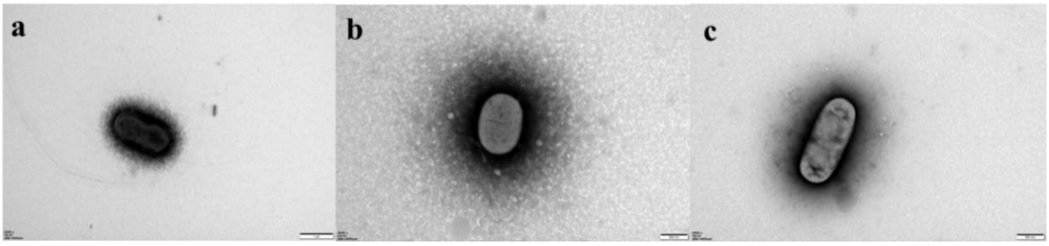


**Figure S1.** Transmission electron microscopy images show the cell morphology of strains SCSIO 75817$^T$ (a), SCSIO 76264$^T$ (b) and SCSIO 80058$^T$ (c) after grown on MA medium at 28 °C for 3 days. **a**: Bar, 1 μm; **b**: Bar, 0.5 μm; **c**: Bar, 0.5 μm.

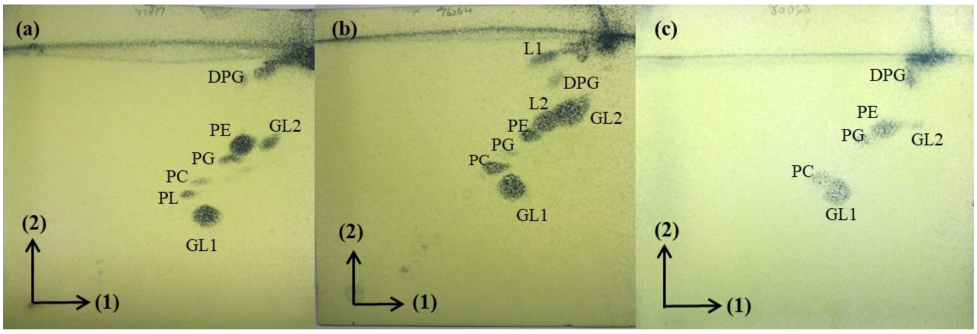


**Figure S2.** Two-dimensional thin-layer chromatography of polar lipids of strains SCSIO 75817$^T$ (a), SCSIO 76264$^T$ (b) and SCSIO 80058$^T$ (c). The first direction developed in chloroform: methanol: water (65:25:4, v/v/v), and the second in chloroform: methanol: acetic acid: water (80:12:15:4, v/v/v/v). DPG, diphosphatidylglycerol; PE, phosphatidylethanolamine; PG, phosphatidylglycerol; PC, phospatidyl choline; PL, unidentified phospholipid; GL, unidentified glycolipid; L, unidentified lipid.

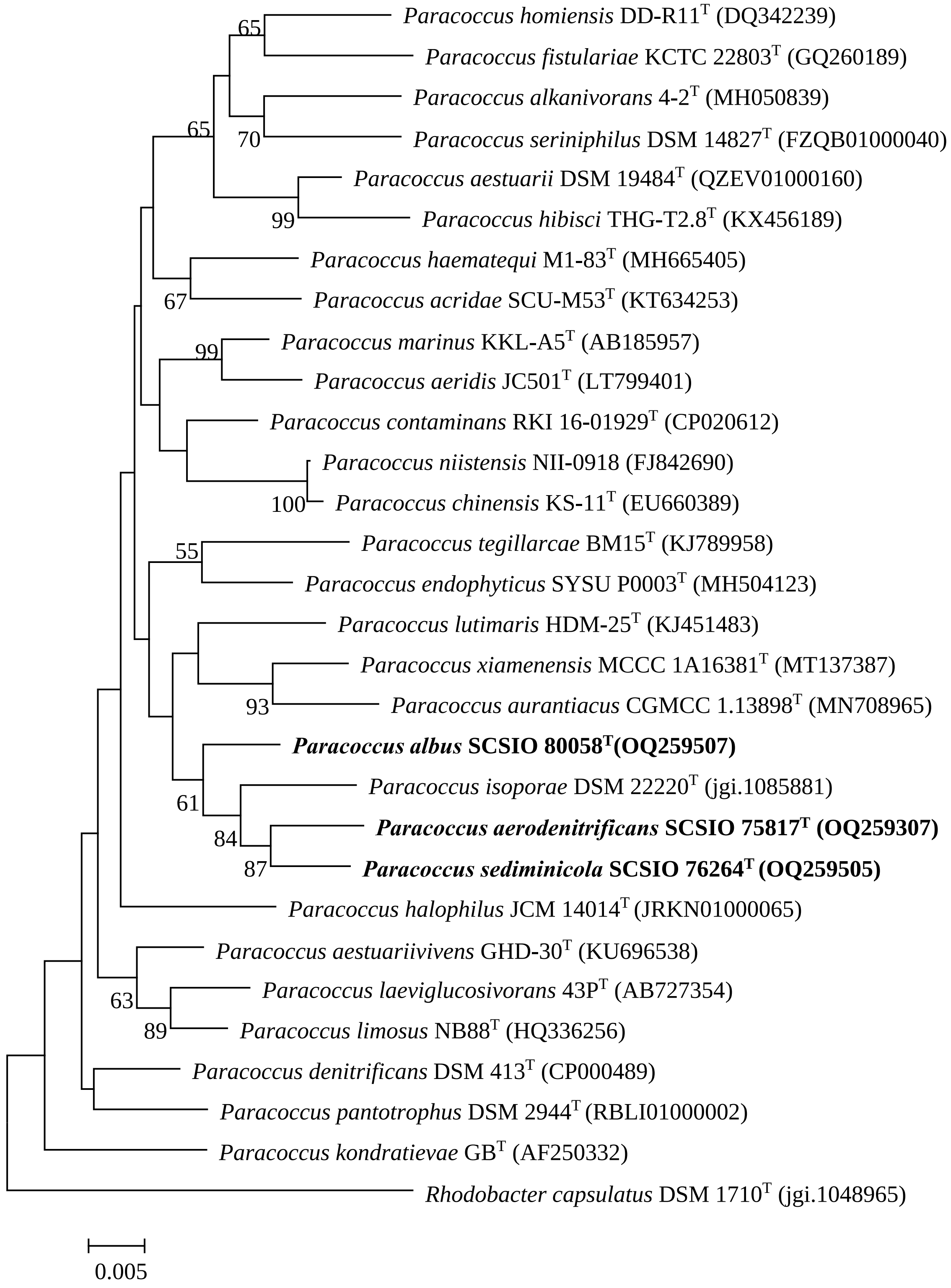


**Figure S3.** Neighbour-joining phylogenetic tree based on nearly complete 16S rRNA gene sequences of strains SCSIO 75817$^T$, SCSIO 76264$^T$ and SCSIO 80058$^T$ with their closest related taxa. *R. capsulatus* DSM 1710$^T$ was used as an outgroup. Numbers at nodes indicate the percentage of 1000 bootstrap replicates. Only bootstrap values above 50% are shown. Bar, 0.005 substitutions per nucleotide position.

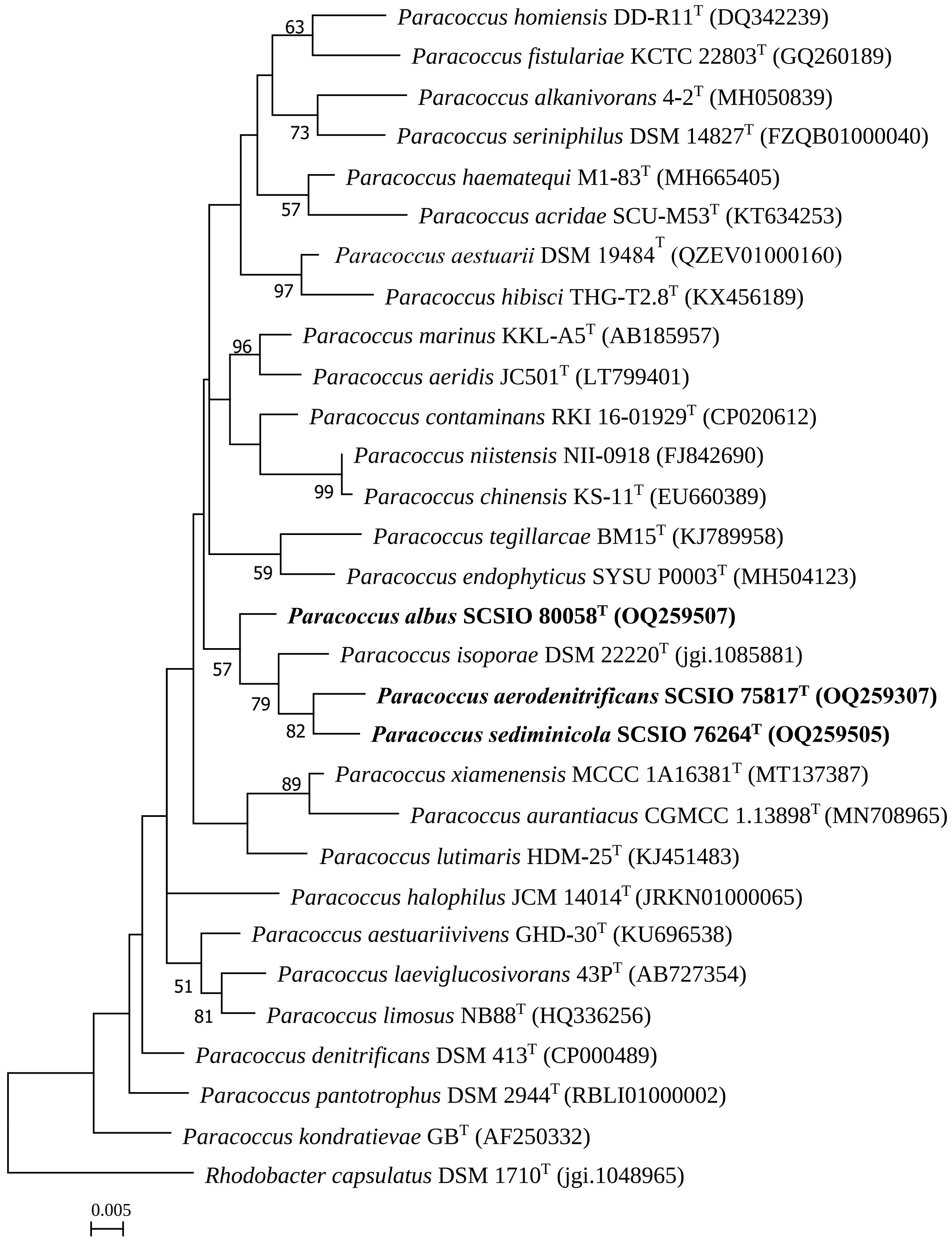


**Figure S4.** The maximum-likelihood phylogenetic tree based on 16S rRNA gene sequences of strains SCSIO 75817$^T$, SCSIO 76264$^T$ and SCSIO 80058$^T$ with related taxa. Numbers at nodes indicate the bootstrap values (> 50%) based on 1000 replications. *Rhodobacter capsulatus* DSM 1710$^T$ (jgi.1048965) was an outgroup. Bar, 0.005 substitutions per nucleotide position.

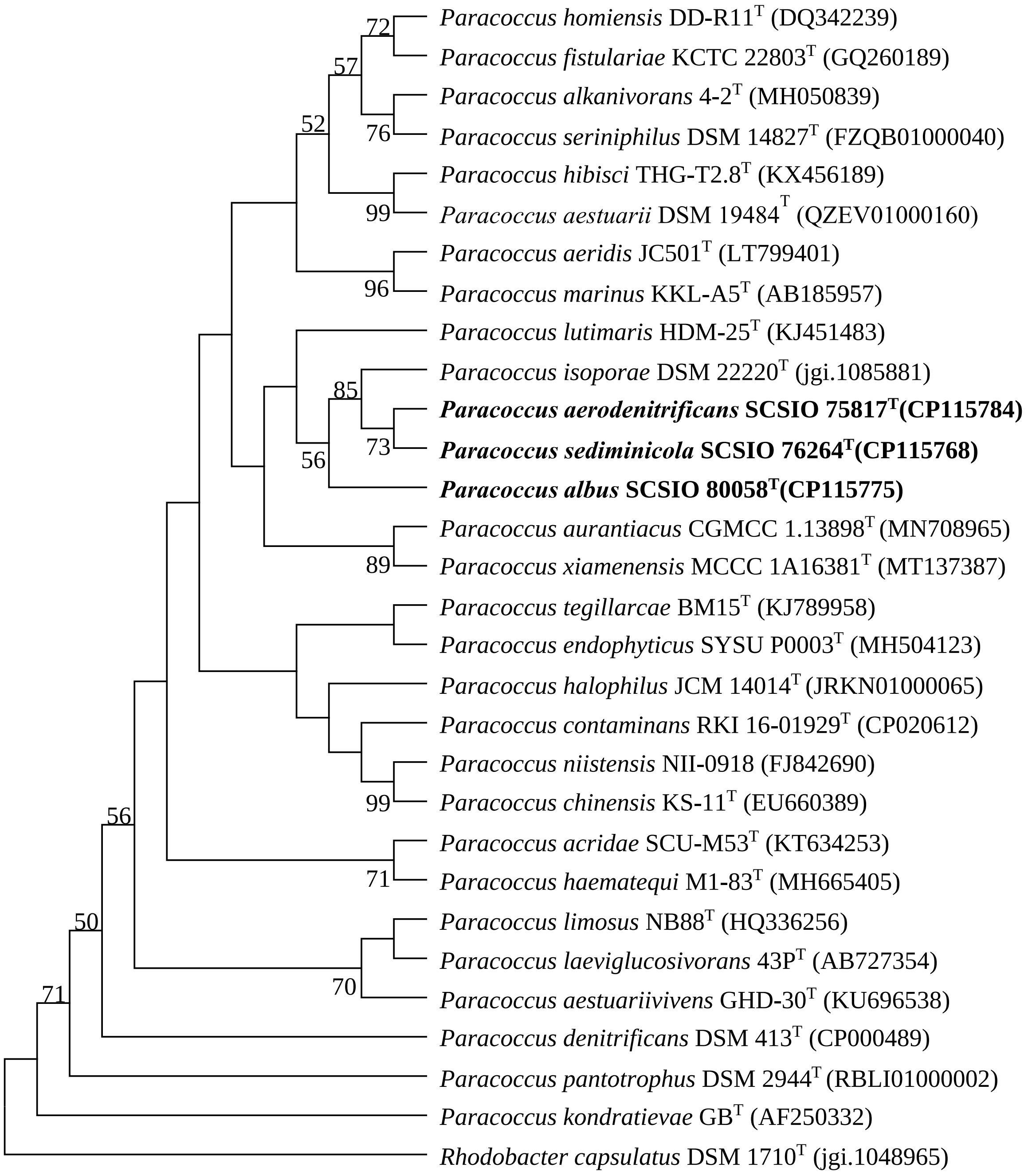


**Figure S5.** The maximum-parsimony phylogenetic tree shows relationships between strains SCSIO 75817$^{T}$, SCSIO 76264$^{T}$, SCSIO 80058$^{T}$ and related taxa based on 16S rRNA gene sequences. Numbers at nodes refer to bootstrap values (1000 replicates; only values > 50% are shown). *Rhodobacter capsulatus* DSM 1710$^{T}$ was used as an outgroup.

**SCSIO 75817$^{T}$**

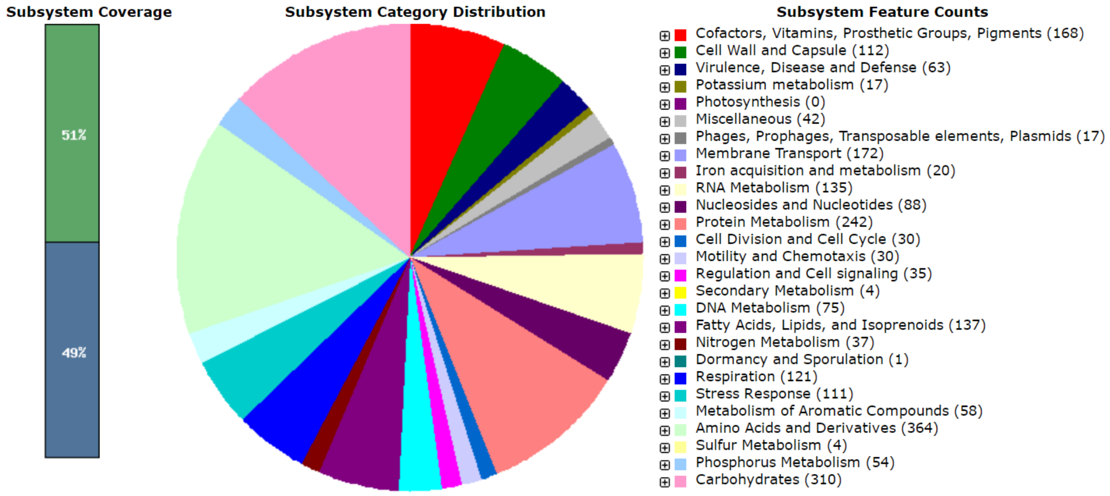


**SCSIO 76264$^{T}$**

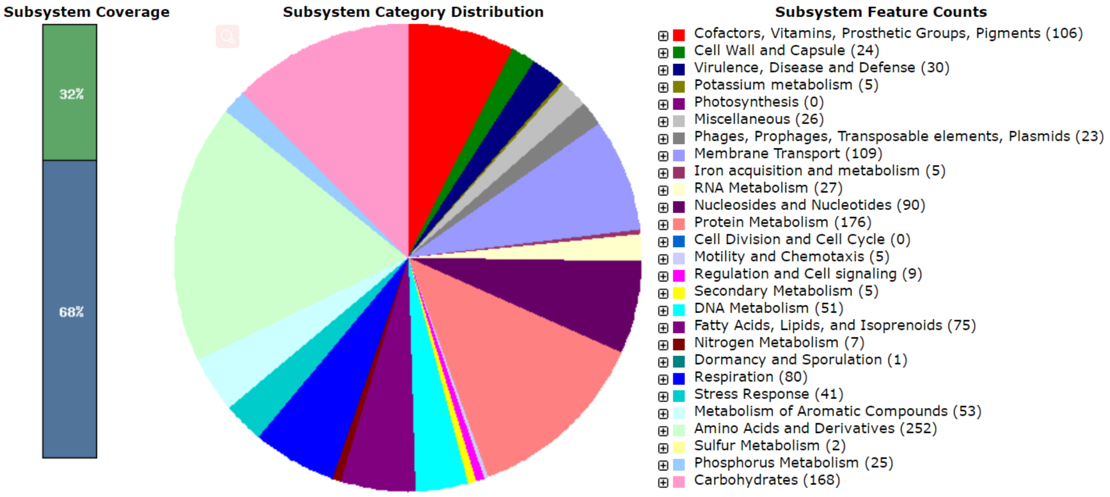


**SCSIO 80058$^{T}$**

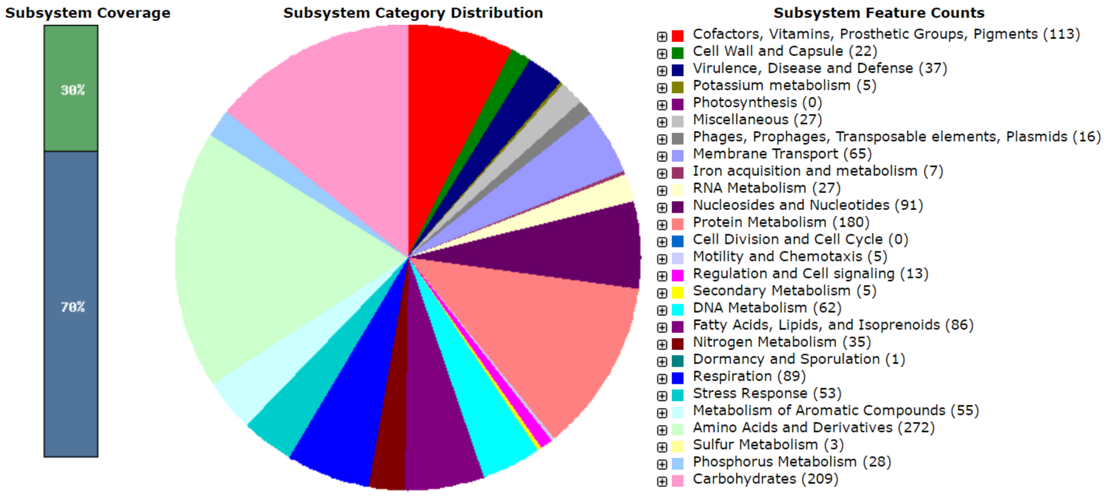


**Figure S6.** The subsystem category number of genes in strains SCSIO 75817$^{T}$, SCSIO 76264$^{T}$ and SCSIO 80058$^{T}$ detected by RAST annotation server.

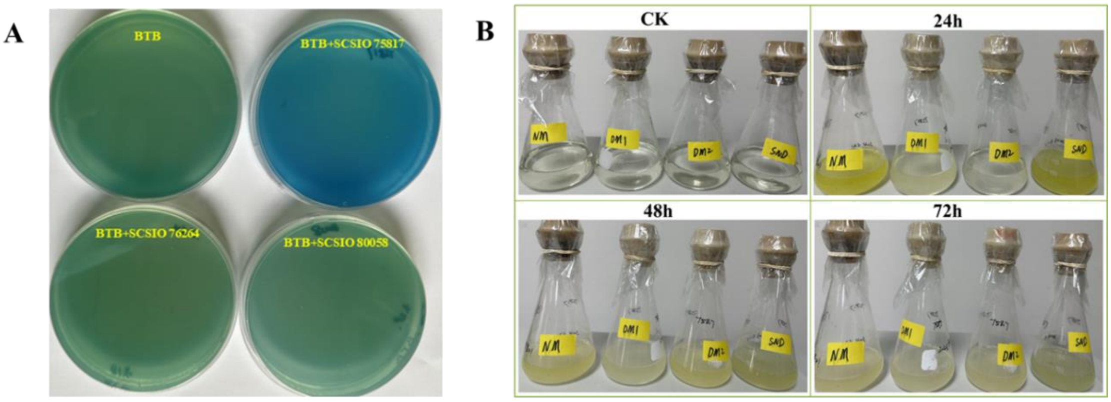


**Figure S7.** Screening of denitrifying bacteria using BTB plate (A) and culture of different sole nitrogen sources (B) including $NH_4^+$-N, $NO_3^-$-N and $NO_2^-$-N.
